# Multi-instance learning of graph neural networks for aqueous p*K*_a_ prediction

**DOI:** 10.1093/bioinformatics/btab714

**Published:** 2021-10-13

**Authors:** Jiacheng Xiong, Zhaojun Li, Guangchao Wang, Zunyun Fu, Feisheng Zhong, Tingyang Xu, Xiaomeng Liu, Ziming Huang, Xiaohong Liu, Kaixian Chen, Hualiang Jiang, Mingyue Zheng

**Affiliations:** Drug Discovery and Design Center, State Key Laboratory of Drug Research, Shanghai Institute of Materia Medica, Chinese Academy of Sciences, Shanghai 201203, China; College of Pharmacy, University of Chinese Academy of Sciences, Beijing 100049, China; Development Department, Suzhou Alphama Biotechnology Co., Ltd, Suzhou City 215000, China; College of Computer and Information Engineering, Dezhou University, Dezhou City 253023, China; Drug Discovery and Design Center, State Key Laboratory of Drug Research, Shanghai Institute of Materia Medica, Chinese Academy of Sciences, Shanghai 201203, China; Drug Discovery and Design Center, State Key Laboratory of Drug Research, Shanghai Institute of Materia Medica, Chinese Academy of Sciences, Shanghai 201203, China; College of Pharmacy, University of Chinese Academy of Sciences, Beijing 100049, China; Tencent AI Lab, Tencent, Shenzhen 518057, China; Drug Discovery and Design Center, State Key Laboratory of Drug Research, Shanghai Institute of Materia Medica, Chinese Academy of Sciences, Shanghai 201203, China; College of Pharmacy, University of Chinese Academy of Sciences, Beijing 100049, China; Drug Discovery and Design Center, State Key Laboratory of Drug Research, Shanghai Institute of Materia Medica, Chinese Academy of Sciences, Shanghai 201203, China; College of Pharmacy, University of Chinese Academy of Sciences, Beijing 100049, China; Drug Discovery and Design Center, State Key Laboratory of Drug Research, Shanghai Institute of Materia Medica, Chinese Academy of Sciences, Shanghai 201203, China; Development Department, Suzhou Alphama Biotechnology Co., Ltd, Suzhou City 215000, China; Shanghai Institute for Advanced Immunochemical Studies, and School of Life Science and Technology, ShanghaiTech University, Shanghai 200031, China; Drug Discovery and Design Center, State Key Laboratory of Drug Research, Shanghai Institute of Materia Medica, Chinese Academy of Sciences, Shanghai 201203, China; College of Pharmacy, University of Chinese Academy of Sciences, Beijing 100049, China; Drug Discovery and Design Center, State Key Laboratory of Drug Research, Shanghai Institute of Materia Medica, Chinese Academy of Sciences, Shanghai 201203, China; College of Pharmacy, University of Chinese Academy of Sciences, Beijing 100049, China; Shanghai Institute for Advanced Immunochemical Studies, and School of Life Science and Technology, ShanghaiTech University, Shanghai 200031, China; Drug Discovery and Design Center, State Key Laboratory of Drug Research, Shanghai Institute of Materia Medica, Chinese Academy of Sciences, Shanghai 201203, China; College of Pharmacy, University of Chinese Academy of Sciences, Beijing 100049, China

## Abstract

**Motivation:**

The acid dissociation constant (p*K*_a_) is a critical parameter to reflect the ionization ability of chemical compounds and is widely applied in a variety of industries. However, the experimental determination of p*K*_a_ is intricate and time-consuming, especially for the exact determination of micro-p*K*_a_ information at the atomic level. Hence, a fast and accurate prediction of p*K*_a_ values of chemical compounds is of broad interest.

**Results:**

Here, we compiled a large-scale p*K*_a_ dataset containing 16 595 compounds with 17 489 p*K*_a_ values. Based on this dataset, a novel p*K*_a_ prediction model, named Graph-p*K*_a_, was established using graph neural networks. Graph-p*K*_a_ performed well on the prediction of macro-p*K*_a_ values, with a mean absolute error around 0.55 and a coefficient of determination around 0.92 on the test dataset. Furthermore, combining multi-instance learning, Graph-p*K*_a_ was also able to automatically deconvolute the predicted macro-p*K*_a_ into discrete micro-p*K*_a_ values.

**Availability and implementation:**

The Graph-p*K*_a_ model is now freely accessible via a web-based interface (https://pka.simm.ac.cn/).

**Supplementary information:**

Supplementary data are available at *Bioinformatics* online.

## 1 Introduction

The acid dissociation constant p*K*_a_, an equilibrium constant defined as the negative logarithm of the ratio of the protonated and deprotonated form of a compound, is a key parameter to describe the ionization ability of substances. It has been reported that about two-thirds of marketed drugs are ionizable in the aqueous solution (Manallack, 2007). Hence, in the design of new drugs, p*K*_a_ is a crucial physical property to be considered, which has profound effects on biological activities, ADMET (absorption, distribution, metabolism, excretion and toxicity) properties and other properties of drugs (Charifson and Walters, 2014; Manallack *et al.*, 2013). Apart from the pharmaceutical industry, the p*K*_a_ is also related to environmental ecotoxicology, agriculture and chemical industries. Hence, the fast and accurate prediction of p*K*_a_ values of chemical compounds from their structures is of great interest.

**Fig. 1.**
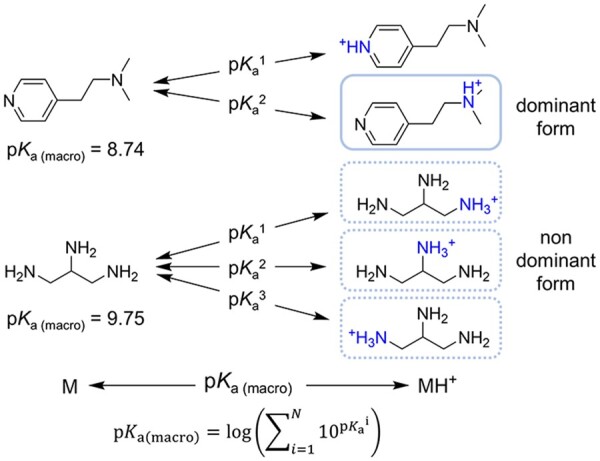
The relationship between macro- and micro-p*K*_a_ of basic compounds. p*K*_a(macro)_ refers to the macro-p*K*_a_; p*K*_a_^1^, p*K*_a_^2^ and p*K*_a_^3^ refer to the micro-p*K*_a_

Graph neural networks (GNN) are a type of neural network to process graph structure data (Defferrard *et al.*, 2016; Niepert *et al.*, 2016). Since first introduced into the prediction of molecular properties several years ago (Duvenaud *et al.*, 2015), reports of different GNN architectures and their successful applications have been rapidly accumulating in this field (Sun *et al.*, 2020; Zhang *et al.*, 2021). However, so far, graph neural networks have rarely been applied in the prediction of p*K*_a_, presumably because the p*K*_a_ values are not only molecular-level ‘global’ properties but also atomic-level ‘local’ properties (Fig. 1). The molecular-level ‘global’ properties refer to the macro-p*K*_a_, the acid dissociation constant related to the observable loss or gain of a proton from a molecule regardless of specific ionization site. The ‘local’ properties refer to micro-p*K*_a_, the acid dissociation constant related to the loss or gain of a proton from a single titratable site (Işık *et al.*, 2018). Apart from the macro-p*K*_a_, a powerful p*K*_a_ prediction model should also be capable of providing micro-p*K*_a_ information at the atomic level. Such information can not only enhance our confidence in the predicted results but also provide useful reference information for the structural modification of compounds, chemical reaction prediction and other related studies. However, for a molecule with multiple ionization sites, usually, we can only measure one or a few macro-p*K*_a_ values experimentally, but not the micro-p*K*_a_ values of all individual sites. Thus, it is intricate to predict micro-p*K*_a_ values, posing a significant challenge to the overall prediction of p*K*_a_.

In 2019, Roszak *et al.* built a graph convolution model for the prediction of the p*K*_a_ value of the C-H bond in organic solvents and applied this model to predict the products of hydrogen abstraction reaction (Roszak *et al.*, 2019). To the best of our knowledge, this study was the only attempt to predict compound p*K*_a_ with graph neural networks. However, the training of their model relied on the p*K*_a_ dataset containing atomic level labels, which were mainly obtained from quantum chemical calculation or molecules with a single ionizable site. Hence, this method is difficult to extend to the p*K*_a_ prediction of heterogeneous chemical classes with multiple ionizable sites. Another alternative strategy to obtain micro-p*K*_a_ data is to assign the macro-p*K*_a_ value of a molecule to its major responsible ionization site and take it as an approximation of the micro-p*K*_a_ value. Recently, some p*K*_a_ prediction models have used this strategy (Hunt *et al.*, 2020; Yang *et al.*, 2020), but there are also two significant problems. As illustrated in Figure 1, (i) for molecules such as propane-1,2,3-triamine, there are multiple sites having similar ionization capacity, this approximate treatment may bring large errors; (ii) the selection of major responsible ionization site is a non-trivial process and requires substantial chemical domain knowledge, and in many cases, a macro-p*K*_a_ value could not be unambiguously assigned to one major ionizable group.

Multi-instance learning (MIL) is a kind of weakly supervised learning algorithm for data with only coarse-grained labels (Zhou, 2018). In classic MIL, the training set is composed of many ‘bags’, each of which contains a series of ‘instances’. A bag is labeled as positive if containing at least one positive instance; otherwise, it is labeled as negative. The goal of MIL is to train a classifier that can correctly label unseen bags. Due to the ability to provide instance-level interpretation, MIL has attracted extensive attention in many classification tasks such as medical image analysis, text classification and video annotation (Carbonneau *et al.*, 2018; Wang *et al.*, 2019; Zhou *et al.*, 2017). However, so far, MIL has rarely been used in regression tasks. This is because a necessary prerequisite for obtaining instance labels through MIL is that there should be a clear mathematical relationship between instance labels and bag labels. This relationship is common in classification tasks (such as ‘or’ relationship) but rare in regression tasks. For p*K*_a_, there is a relatively clear relationship between macro-p*K*_a_ and micro-p*K*_a_. For example, Figure 1 shows the formula between macro-p*K*_a_ and micro-p*K*_a_ of basic compounds.

**Fig. 2.**
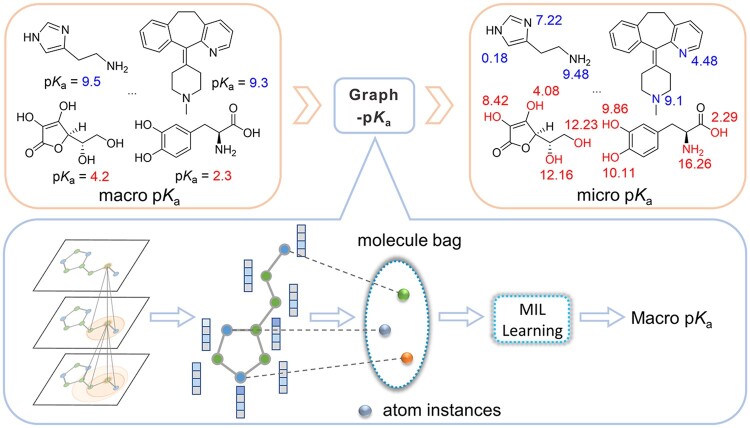
The schematic representation of the proposed Graph-p*K*_a_ model

Here, combining multi-instance learning and graph neural networks, we designed a novel p*K*_a_ prediction model named Graph-p*K*_a_. In Graph-p*K*_a_, a molecule is regarded as a ‘bag’, and those ionizable atoms in this molecule are regarded as ‘instances’. It means that the macro-p*K*_a_ value of a molecule is designated as the label of a bag, which is available in the training set, and the unavailable information regarding to the micro-p*K*_a_ values of ionizable sites are considered as the labels of instances. Under this scheme, Graph-p*K*_a_ can follow the MIL framework to learn the labels of instances through training against the labels of bags (Fig. 2). Furthermore, it should be noted that those molecules containing multiple ionization sites may have multiple macro-p*K*_a_ values. In this work, we only consider the most acidic and basic p*K*_a_ values, which are key parameters that can unambiguously and concisely describe the ionization capabilities of compounds. Some chemical information websites, including ChEMBL (Gaulton *et al.*, 2017) and DrugBank (Wishart *et al.*, 2018) also describe the prediction for the p*K*_a_ of compounds in terms of the most acidic and basic p*K*_a_ values.

## 2 Materials and methods

### 2.1 S-p*k*_a_ dataset

**Fig. 3.**
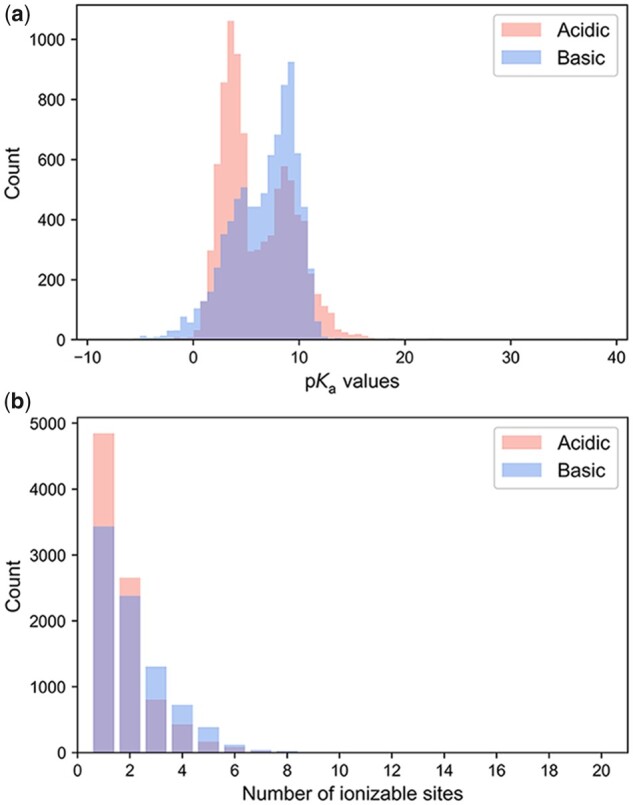
The distributions of simple compound properties in the S-p*K*_a_ dataset. (**a**) The experimental p*K*_a_ values. (**b**) The number of ionizable sites

A large p*K*_a_ dataset named S-p*K*_a_ was compiled, mainly from three main sources: (i) datasets used in several previous studies on p*K*_a_, (ii) a free software named QSAR Toolbox, (iii) manual extraction from various literature. Those chemical structures from different sources were standardized and then merged. The structure standardization procedure includes removing all salts from molecules, neutralizing charged molecules, and standardizing SMILES strings. In addition, considering that the accuracy of publicly available experimentally determined p*K*_a_ values was often dubious (Rupp *et al.*, 2011), each data would undergo manual inspection to ensure that it belongs to the most acidic or basic p*K*_a_ value of its corresponding molecule before adding to the S-p*K*_a_ dataset. The detailed processes of data collection and cleaning is given in Supplementary Material and Supplementary Figure S1. The S-p*K*_a_ dataset can be separated into an acidic subset and a basic subset, containing the most acidic p*K*_a_ values of 9043 chemical structures and the most basic p*K*_a_ values of 8436 chemical structures, respectively (Supplementary Fig. S2a). The distribution of p*K*_a_ values in the acidic and basic subset is shown in Figure 3a. The most acidic p*K*_a_ values varied from -3.3 to 40, while the most basic p*K*_a_ values varied from -10.1 to 14. Since to learn micro-p*K*_a_ via MIL is a critical concept utilized in the establishment of the Graph-p*K*_a_ model, the acidic or basic ionizable sites of compounds in the S-p*K*_a_ dataset are all enumerated and displayed (Fig. 3b). In this study, the acidic ionizable sites are defined as non-carbon atoms connected with at least one hydrogen atom, and the basic ionizable sites are defined as nitrogen atoms with no positive formal charge. The distribution of the molecular weight of compounds across the S-p*K*_a_ dataset is also shown in Supplementary Figure S2b.

### 2.2 Graph-p*K*_a_ model

The architecture of Graph-p*K*_a_ is shown in Figure 2. It begins by describing each molecule as an undirected graph where nodes and edges correspond to atoms and chemical bonds, respectively. The molecular graph is then input into the graph neural layers where atoms receive the message of other atoms in the molecule and use the aggregated messages to update their own features. The graph neural layer in Graph-p*K*_a_ is the same as our previously developed Attentive FP (Xiong *et al.*, 2020), a molecular representation learning scheme that uses a graph attention mechanism. Here, six graph neural layers are stacked in Graph-p*K*_a_ for the extraction of atom features.

The major difference between Graph-p*K*_a_ and other graph neural networks lies in the approach to deal with the features of nodes extracted by graph neural network layers. In molecular graph neural networks such as GCN (Duvenaud *et al.*, 2015), MPNN (Gilmer *et al.*, 2017) and Attentive FP (Xiong *et al.*, 2020), those node features are aggregated with various pooling operations such as average pooling and Set2Set to generate the features of the whole molecule, which are next used to fit and predict the molecular properties. However, in Graph-p*K*_a_ those learned node features are directly fed into a fully connected (FC) layer to predict the p*K*_a_ values of atoms. Since some atoms in molecules are not ionizable, their predicted p*K*_a_ values will be masked. In the acidic and basic p*K*_a_ prediction model, the mask values are respectively positive infinity and negative infinity. Finally, the macro-p*K*_a_ values of molecules are calculated according to the approximate mathematical relationships between them and the predicted p*K*_a_ values of ionizable atoms. More specifically, given an atom *A_i_* with features *X_i_*, the above process can be formulated as follows:

In acidic p*K*_a_ prediction model:

(1) $${{pK}_{a\left(\mathrm{acidic}\right)}}^{i}=\mathrm{FC}\left(X_{i}\right)$$

 

(2) $${{pK}_{a\left(\mathrm{acidic}\right)}}^{i}=\left\{\begin{array}{l} {{pK}_{a(\mathrm{acidic})}}^{i},\;A^{i}\in P\\ inf,\;A^{i}\notin P \end{array}\right.$$

 

(3) $${pK}_{a\left(\mathrm{acidic}\right)}=-\log\left(\sum_{i=1}^{N}10^{{{-pK}_{a\left(\mathrm{acidic}\right)}}^{i}}\right)$$

In basic p*K*_a_ prediction model:

(4) $${{pK}_{a\left(\mathrm{basic}\right)}}^{i}=\mathrm{FC}\left(X_{i}\right)$$

 

(5) $${{pK}_{a\left(\mathrm{basic}\right)}}^{i}=\left\{\begin{array}{l} {{pK}_{a(\mathrm{basic})}}^{i},\;A^{i}\in Q\\ -\inf,\;A^{i}\notin Q \end{array}\right.$$

 

(6) $${pK}_{a\left(\mathrm{basic}\right)}=\log\left(\sum_{i=1}^{N}10^{{{pK}_{a\left(\mathrm{basic}\right)}}^{i}}\right)$$

where FC is referred to a fully connected neural network layer, *P* is the acidic ionizable sites, *Q* is the basic ionizable sites, N is the number of heavy atoms in a molecule, inf is the positive infinity, ${pK}_{a(\mathrm{acidic})}$ and ${pK}_{a(\mathrm{basic})}$ are the most acidic/basic p*K*_a_ values of a molecule.

Obviously, formula 3 and 6 are the key formulas for MIL. Yang *et al.* also had used formula 6 to calculate the macro-p*K*_a_ values in their study (Yang *et al.*, 2020). Here, we provided the derivation of formula 3 and 6 in Supplementary Material and Supplementary Figure S3.

### 2.3 Implement of Graph-p*K*_a_ and other benchmark methods

In Graph-p*K*_a_, the conversion from a SMILES string to an undirected graph and initialization for it was implemented with the DGL-LifeSci package. The representations of the graph were initialized with eight kinds of atom features and four kinds of bond features (Supplementary Table S1). The Graph-p*K*_a_ model was implemented using the PyTorch and DGL. The loss function used to train Graph-p*K*_a_ was MSELoss. Attentive FP and four machine learning models, including SVM, RF, XGBoost and ANN were implemented as baseline models. XGBoost was implemented with the XGBoost package, SVM, RF and ANN were implemented with the Scikit-learn package. Attentive FP is a graph neural network with the same GNN layers as Graph-p*K*_a_ but without MIL, which was also implemented as a control for model performance evaluation. For baseline models except Attentive FP, the molecular fingerprints used to encode the molecular structures were a kind of combined molecular fingerprint that integrated eight types of common molecular fingerprints including CDK, Estate, CDK graph only, MACCS, PubChem, Substructure, Klekota-Roth and 2D atom pairs. Those molecular fingerprints had 9121 bits in total and were calculated using PaDEL(Yap, 2011).

### 2.4 Model training and evaluation

In the experiment of predicting macro-p*K*_a_, the S-p*K*_a_ dataset was randomly split into training/validation/test set in a 70:15:15 ratio. Graph-p*K*_a_ and other models were trained on the same training set. The best set of hyperparameters for each model were determined based on the result on the validation set. The search ranges and optimal values of these hyperparameters are provided in Supplementary Table S2. The final model performance was assessed on the test set and two external tests set through three independent runs. The metrics for evaluating model performance were mean absolute error (MAE), root mean squared error (RMSE) and coefficient of determination (*R*^2^). The structural similarity between the two molecules was calculated using the 1024-bit Morgan2 fingerprints and the Tanimoto coefficient.

In the experiment of predicting micro-p*K*_a_, about 500 molecules that possessed multiple different acidic/basic ionization sites and whose dominant ionization sites had been uniquely assigned by Hunt *et al.* (2020) were extracted as test data. Those molecules were then removed from the S-p*K*_a_ Dataset. Graph-p*K*_a_ was retrained on the remaining dataset with the same set of hyperparameters previously used. The metrics for evaluating the model are consistency rate and difference values. Consistency rate is the probability that the dominant ionization sites of molecules selected by Graph-p*K*_a_ are the same as that of human experts. Different value is used to quantify the degree of divergence between Graph-p*K*_a_ and human experts. They are calculated as follows:

(7) ci=1, hi∈Gi0, hi∉Gi

 

(8) $$\mathrm{consistency}\;\mathrm{rate}=\;\frac{1}{n}\sum_{i=1}^{n}ci$$

 

(9) $$\mathrm{difference}\;\mathrm{value}=\mathrm{Abs}\left(f\left(g_{i}\right)-f\left(h_{i}\right)\right)$$

where $h_{i}$ is the most acidic/basic atom of molecule i selected by human experts, $G_{i}$ is the most acidic/basic atoms of molecule i predicted by Graph-p*K*_a_, $g_{i}\;$is an arbitrary element in $G_{i}$, the reason why $G_{i}$ is a collection is that some molecules have multiple dominant ionization sites with the same ionization ability, f is referred to a function of Graph-p*K*_a_ for atomic p*K*_a_ prediction.

## 3 Results and discussion

### 3.1 Comparison with benchmark methods

**Fig. 4.**
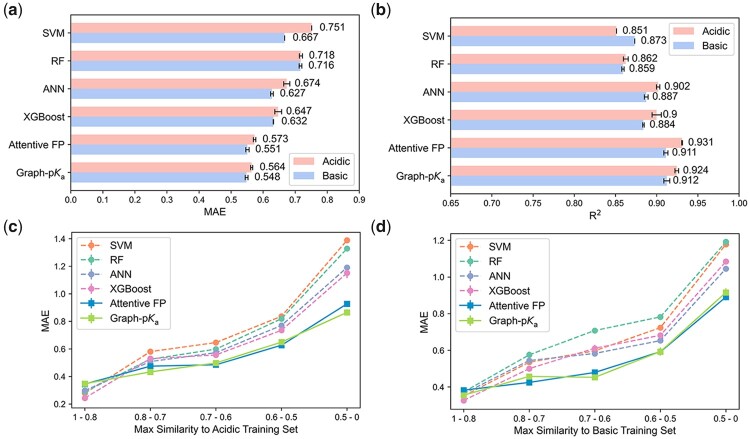
The performance of the various model on macro-p*K*_a_ prediction on the S-p*K*_a_ dataset. (**a,b**) The MAE and R^2^ of those models on the test dataset. (**c,d**) The MAE of those models for acidic (c) and basic (d) p*K*_a_ prediction on a series of similarity subsets. Error bars represent standard deviations

In order to evaluate the performance of Graph-p*K*_a_, four conventional machine learning models were implemented and taken as benchmark methods. A kind of combined molecular fingerprints was used as the representation of molecules and the input of these machine learning models, due to its good performance on a previous study for p*K*_a_ prediction (Mansouri *et al.*, 2019). The comparison between Graph-p*K*_a_ and other models was carried out on the S-p*K*_a_ dataset that was randomly divided into training, validation and test set. The performances of those models on the test set are shown in Figure 4. Among the four machine learning models, ANN and XGBoost performed comparatively well, which was consistent with some previous studies (Mansouri *et al.*, 2019; Yang *et al.*, 2020). However, the performances of these two models still obviously fell behind Graph-p*K*_a_, which achieved a MAE around 0.55 and a *R*^2^ around 0.92 on the test sets (Fig. 4a and b). As known, the performance of QSAR models is closely related to the similarity between predicted molecules and the molecules of the training set. To evaluate the generalization capability of different models, we also calculated the pairwise similarity of test set molecules to the training set molecules, and split the test set molecules into five individual subsets according to their maximum similarity to training set molecules (Supplementary Table S3). Then, the MAE of those models on each subset was compared. As shown in Figure 4c and d, the Graph-p*K*_a_ outperformed other machine learning models on nearly all similarity subsets, which demonstrated it possesses high robustness and generalization ability. For the molecules with max similarity higher than 0.5 to the training set, the MAE of the model was lower than 0.65. If using it as the threshold for acceptable errors, 81.1% of test molecules were within the applicability domain of the models. Furthermore, the performance of Attentive FP on macro-p*K*_a_ prediction was not better than that of Graph-p*K*_a_, meaning that MIL could endow Graph-p*K*_a_ with the prediction ability of micro-p*K*_a_ without significant trade-off on its prediction ability of macro-p*K*_a_.

### 3.2 Evaluation on external datasets

The performance of Graph-p*K*_a_ was further validated by testing against two external datasets that were obtained from two blind p*K*_a_ prediction challenges named SAMPL6 and SAMPL7. These two challenges were launched by the Drug Design Data Resource Community in 2018 and 2020, respectively. The SAMPL6 dataset comprises 24 kinase inhibitor-like molecules with 31 experimental p*K*_a_ values, and the SAMPL7 dataset comprises 22 molecules (most are sulfonamides) with 20 experimental p*K*_a_ values. There are two p*K*_a_ values not belonging to the most acidic or basic p*K*_a_ values in the SAMPL6 dataset and two molecules without corresponding experiment p*K*_a_ values in the SAMPL7 dataset, they were excluded from this testing. The performances of Graph-p*K*_a_ and some commonly used software and models on these two external datasets are shown in Table 1 and Supplementary Table S4. Graph-p*K*_a_ achieved a low MAE of 0.594 and 0.758 as well as a high R^2^ of 0.918 and 0.839 on SAMPL6 and SAMPL7 datasets, respectively, comparable to the performance of those commercial software established based on large collections of proprietary data.

**Table 1.** Performance of Graph-p*K*_a_ and other models on the SAMPL6 and SAMPL7 external test sets

Dataset	Model name	Model class	MAE	RMSE	*R* ^2^
SAMPL6	Epik Scan^a^	Commercial	0.784	0.962	0.857
	Epik Micro^a^	Commercial	0.783	0.972	0.854
	ACD/pKa^a^	Commercial	**0.550**	0.783	0.905
	MoKa^a^	Commercial	0.854	0.970	0.854
	ChemAxon^a^	Commercial	1.007	1.248	0.759
	Hunt’s model^b^	Academic	0.687	0.864	0.885
	Yang’s XGB^b^	Academic	0.767	1.011	0.842
	Yang’s NN^b^	Academic	0.832	1.141	0.799
	OPERA^d^	Academic	0.970	1.283	0.619
	Graph-p*K*_a_	Academic	0.594	**0.726**	**0.918**
SAMPL7	Epik Scan^c^	Commercial	1.121	1.648	0.508
	ChemAxon^c^	Commercial	**0.559**	**0.708**	**0.909**
	Yang’s XGB^c^	Academic	1.476	1.622	0.523
	Yang’s NN^c^	Academic	0.932	1.156	0.758
	OPERA^d^	Academic	2.135	2.515	−3.752
	Graph-p*K*_a_	Academic	0.758	0.934	0.839

The bold entries in the “MAE”, “RMSE”, and “R2” columns represent the best results in corresponding datasets.

a The results are cited from a summary of the SAMPL6 challenge results. (https://github.com/samplchallenges/SAMPL6/blob/master/physical_properties/pKa/analysis/).

b The results are cited from articles of Hunt *et al.* (2020) and Yang *et al.* (2020).

c The results of Epik predictions are from Schrödinger Suite 2017; the results of ChemAxon predictions are from ChemAxon Marvin Suite 20.15.0. The results of Yang’s XGB and Yang’s NN are from a webserver (http://pka.luoszgroup.com/prediction).

d The results are from OPERA 2.7. Nine p*K*_a_ values that OPERA2.7 failed to predict were excluded.

Although our Graph-p*K*_a_ model has achieved satisfactory prediction performance, there are potentially two limitations. Frist, Graph-p*K*_a_ is only trained to predict the most acidic and basic p*K*_a_ values and its capability to predict other types of p*K*_a_ values such as the 2nd strongest acidic and basic p*K*_a_ values has not been fully evaluated. This is mainly because of the difficulty in the collection, cleaning, and labeling of this kind of training data. Second, the tautomerism of molecules has not been taken into account in Graph-p*K*_a_, which means that the model will give different prediction results for different tautomers of the same molecule. We leave this issue to follow-up studies, such as averaging the predicted values of different tautomers.

### 3.3 Performance on micro-p*K*_a_ prediction

**Fig. 5.**
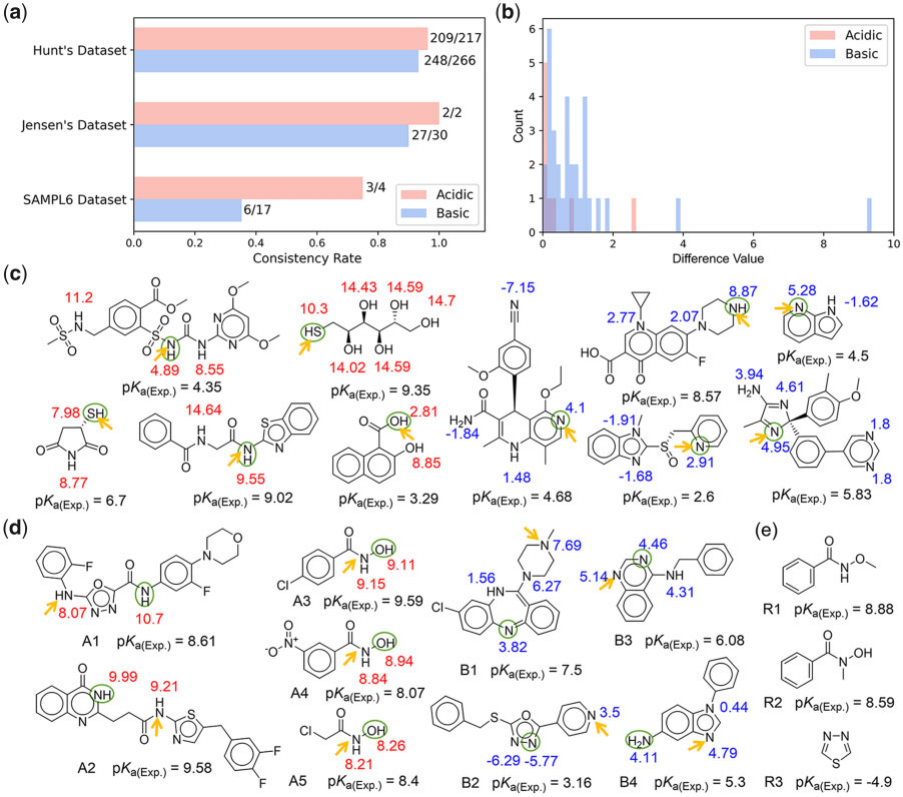
Application of Graph-p*K*_a_ to predict the dominant ionization sites of molecules. (**a**) The consistency rates between the prediction of Graph-p*K*_a_ and the judgment of human experts. (**b**) The distribution of difference values representing the degree of divergence between Graph-p*K*_a_ and human experts on controversial molecules. (**c,d**) Some examples of molecules on which the predictions of Graph-p*K*_a_ and human experts are consistent (c) and different (d), the arrows and circles denote to the dominant ionization sites selected by Graph-p*K*_a_ and human experts, respectively, red and blue numbers, respectively, denote to the predicted acidic and basic p*K*_a_ values of atoms by Graph-p*K*_a_. (**e**) Some molecules and their p*K*_a_ values for reference

Macro-p*K*_a_ values can describe the ionization degree of the molecule in the solvent but can't pinpoint the ionization state of each atom in this molecule. To acquire more comprehensive knowledge about the ionization of molecules, the prediction of micro-p*K*_a_ values is equally important. Thus, the performance of Graph-p*K*_a_ on predicting micro-p*K*_a_ was also evaluated here. Unfortunately, the experimental determination of micro-p*K*_a_ values is highly complicated, and there is currently no available micro-p*K*_a_ dataset. Given this situation, a Turing-like test was designed to determine if Graph-p*K*_a_ exhibited the intelligent behavior (i.e. to designate the most acidic/basic atoms in a molecule structure) that was indistinguishable from that of a human expert. The results of expert judgments were obtained from a recent work of Hunt *et al.* for p*K*_a_ prediction (Hunt *et al.*, 2020), where each p*K*_a_ value in their collected dataset (Hunt’s dataset) and two external test sets (Jensen’s dataset and SAMLP6 dataset) was carefully inspected and assigned to a specific site by human experts. As shown in Figure 5a, the overall consistency rates between the most acidic/basic atoms predicted by Graph-p*K*_a_ and the most acidic/basic atoms selected by the human experts were over 90%. To further quantify the degree of divergence between Graph-p*K*_a_ and human experts on those controversial molecules, the difference values of the predicted p*K*_a_ between the most acidic/basic atoms predicted by Graph-p*K*_a_ and those selected by the human experts are shown in Figure 5b. It could be observed that the difference values of 80% these controversial molecules were within 1.2 p*K*_a_ units, which indicated that the divergences between Graph-p*K*_a_ and human expert mainly derived from those molecules whose several atoms had similar ionization capability.

Some examples of agreement and disagreement between Graph-p*K*_a_ and human experts are respectively shown in Figure 5c and d. In the assignment of the most acidic atoms, two controversial molecules of note were A1 and A2, and most of the others were hydroxamic acid derivatives. Hunt *et al.* attributed the acidities of hydroxamic acid derivatives all to their hydroxyls. In fact, the dissociation ability of hydroxylic hydrogen and amino hydrogen in hydroxamic acids was quite similar (Bartmess, 2010) (also see R1, R2 in Fig. 5e, http://ibond.nankai.edu.cn), and the prediction results of Graph-p*K*_a_ supported their equivalent protonation potential. In the assignment of the most basic atoms, the two most controversial molecules were B1 and B2. Our prediction for B1 was supported by a record from PubChem that the p*K*_a_ value of the amine in B1 was 7.75 (https://pubchem.ncbi.nlm.nih.gov/compound/135398737). In addition, the basicity of the 1,3,4-Oxadiazol ring in B2 should be very weak, given that the p*K*_a_ of 1,3,4-thiadiazole was only -4.9 (R3 in Fig. 5e, https://www.scripps.edu/baran/heterocycles/Essentials1-2009.pdf). According to Graph-p*K*_a_ prediction, the basicity of B2 was attributed to the pyridine ring, instead of the 1,3,4-Oxadiazol ring. This assignment was further confirmed by quantum chemical calculation. As shown in Supplementary Figure S4, the protonation energies of nitrogen atom in the pyridine ring were -5.25 kcal/mol, significantly lower than that of nitrogen atoms in the 1,3,4-Oxadiazol ring (4.74 and 5.39 kcal/mol). The methods of quantum chemistry calculation are described in Supplementary Material. Besides, two molecules (B3, B4) in SAMPL6 datasets (Işık *et al.*, 2018), whose dominant ionization sites have been determined by nuclear magnetic resonance, are also shown in Figure 5d. The predicted results of Graph-p*K*_a_ were consistent with the experimental results. The above results demonstrated that Graph-p*K*_a_ performed outstandingly in the prediction of micro-p*K*_a_. It is impressive that in many cases the capability of Graph-p*K*_a_ to locate the most acidic/basic sites of molecules is equivalent to or better than that of human experts, while all the chemical insight has been learned without explicit supervision in multi-instance learning. It can be expected that when there are more available training data in the future, the capability will be further improved.

### 3.4 Visualization of the atomic embeddings

**Fig. 6.**
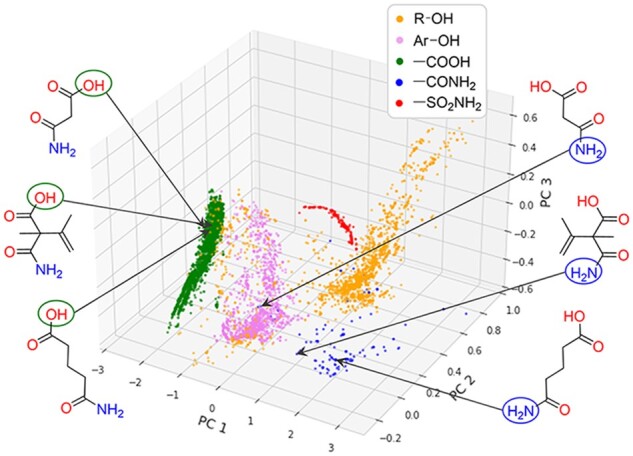
Visualizing the atomic embeddings in last hidden layer using principal component analysis

In order to visualize the features of the atoms learned by the Graph-p*K*_a_ model, the embeddings in the last hidden layer of several types of acidic ionization sites in the training data were extracted and submitted to principal component analysis. As shown in Figure 6, after training, the atomic embeddings from phenol hydroxyl, carboxyl, and sulfonamide groups were respectively gathered together. However, the distributions of atomic embeddings from alcoholic hydroxyl and amide groups were still relatively dispersed. These patterns suggest that alcoholic hydroxyl or amide groups in different chemical environments exhibit relatively larger variances, posing challenges for accurate micro-p*K*_a_ prediction. We speculated that a possible reason was that, although alcoholic hydroxyl and amide groups widely existed in the training set, they have less contribution to the macro-p*K*_a_ of the whole molecule due to their weak acidity. Therefore, they had lower weights and were less supervised during model training. Three molecules and their atomic embeddings visually display such a situation. After training, the atomic embeddings from carboxyl groups of the three similar molecules are close, whereas the atomic embeddings from amide groups of the three molecules are dispersed. Apparently, adding more samples whose dominant ionization groups are alcoholic hydroxyl groups or amide groups into training data may alleviate this problem.

### 3.5 Web server for the prediction of p*K*_a_

For the convenience of the community, a free web server wrapping the Graph-p*K*_a_ model has been developed (https://pka.simm.ac.cn/). This web server was built using the python language and could be simultaneously accessed by multiple users. The web server can take multiple types of inputs including drawing a molecule from the molecular editor or uploading a txt/mol/sdf file. There are two main functions in this web server: p*K*_a_ prediction and similarity search (Supplementary Fig. S5). In the p*K*_a_ prediction module, the most acidic/basic p*K*_a_ values and their corresponding micro-p*K*_a_ values of the input molecule are predicted. The Monte Carlo dropout is used to evaluate the uncertainty of the prediction results and calculate the 95% confidence interval of the predicted value (Gal and Ghahramani, 2016). It is noteworthy that, due to our definition of possible ionization sites and the processing of input molecules, the web server does not support the p*K*_a_ prediction for C-H bonds and ionized molecules. In the similarity search module, the most acidic/basic atoms of the molecules from the S-p*K*_a_ dataset and the most acidic/basic atoms of the molecule input by the user are first predicted by Graph-p*K*_a_. Then, the embeddings of those predicted most acidic/basic atoms in the last hidden layer are extracted. Finally, the Euclidean distances between the atomic embeddings of the input molecule and that of the molecules in the S-p*K*_a_ dataset are calculated. If the Euclidean distance is close enough (the threshold is set as less than 0.05), molecules are considered to be similar, and for each input molecule, up to four similar molecules and their experimentally determined p*K*_a_ values will be output for reference.

## 4 Conclusions

In this work, we have developed a novel in silico p*K*_a_ prediction model named Graph-p*K*_a_. Combining multi-instance learning into graph neural network, Graph-p*K*_a_ not only outperforms those conventional machine learning models based on molecular fingerprints in predicting macro-p*K*_a_, but more significantly, can learn the micro-p*K*_a_ values of atoms through training against the macro-p*K*_a_ values of molecules. A Turing-like test demonstrated that it gained chemical insights to locate the most acidic/basic sites of molecules, which compared favorably with that of human experts. Such micro-p*K*_a_ inference ability greatly enhances the interpretability and practicability of this model. Furthermore, in Graph-p*K*_a_, the fitting and prediction of macro-p*K*_a_ are all dependent on the reasoning of micro-p*K*_a_, which can also avoid shortcut learning to some extent (Geirhos *et al.*, 2020). In the end, a Web application based on Graph-p*K*_a_ model has been made freely available at https://pka.simm.ac.cn.

## Funding

This work was supported by the Project supported by Shanghai Municipal Science and Technology Major Project, National Natural Science Foundation of China [81773634] and Tencent AI Lab Rhino-Bird Focused Research Program [JR202002].

*Conflict of Interest*: none declared. 

## Supplementary Material

btab714_Supplementary_Data
