# Supplementary material for: Multi-instance learning of graph neural networks for aqueous pKa prediction
Source: Bioinformatics. 2021 Oct 13;38(3):792–8. doi: 10.1093/bioinformatics/btab714 (PMC8756178; doi:10.1093/bioinformatics/btab714)
Supplement: btab714_Supplementary_Data [file btab714_supplementary_data.docx]

**Supporting Information**

Multi-instance learning of graph convolutional networks for

aqueous p*K*_a_ prediction

Jiacheng Xiong, Zhaojun Li, Guangchao Wang, Zunyun Fu, Feisheng Zhong, Tingyang Xu, Xiaomeng Liu, Ziming Huang, Xiaohong Liu, Kaixian Chen, Hualiang Jiang*, and Mingyue Zheng*

**The detailed processes of data collection and cleaning**

The S-p*K*_a_ dataset was collected from three main sources: (1) datasets used in several previous studies on p*K*_a_, (2) a free software named QSAR Toolbox, (3) manual extraction from various literature. Before data merging, all chemical structures in those data were first standardized with the RDKit python package. The structure standardization procedure included removing all salts from molecules, neutralizing charged molecules, and standardizing SMILES strings. The p*K*_a_ values not belonging to the most acidic or basic p*K*_a_ were discarded. For the molecule having multiple records of p*K*_a_ values in one data source, we removed those apparent outliers and calculated the median of remaining values as its final p*K*_a_ value. Besides, in consideration of the difference in reliability of p*K*_a_ data from various sources, these data were added into S-p*K*_a_ dataset in a certain order. For example, the p*K*_a_ data used in the study of Hunt et al. had been inspected and cleaned by them. Hence, it was regarded as the most reliable data source and first added into S-p*K*_a_ dataset. Each data was further inspected by us to ensure that it belonged to the most acidic or basic p*K*_a_ value of its corresponding molecule before it was added to the S-p*K*_a_ dataset. Once a piece of data had been checked and added into the S-p*K*_a_ dataset, those data with the same structure and p*K*_a_ type from other sources would be discarded. In S-p*K*_a_ dataset, there is no molecule identical to molecules in other external test sets. The sources of data and their orders when merged into S-p*K*_a_ dataset are shown in Fig S1.


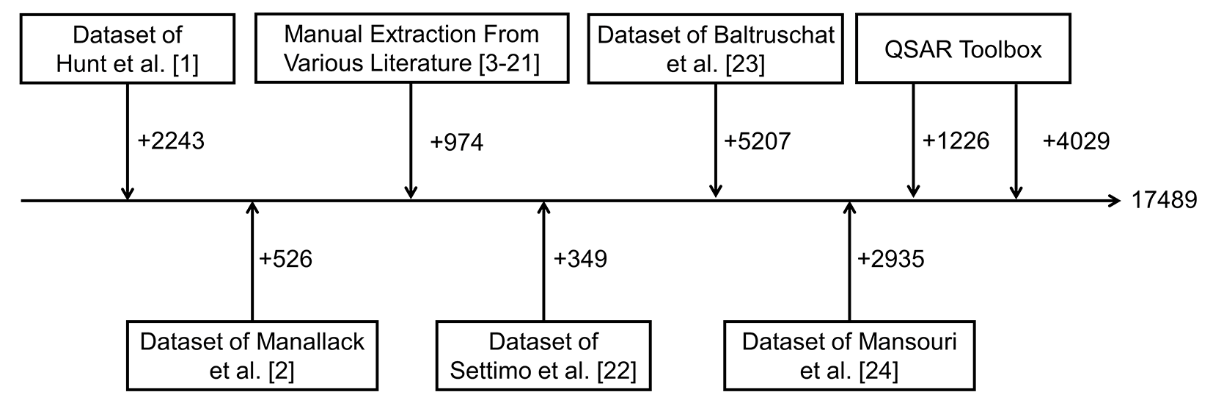


**Fig. S1.** The sources of data and their orders when merged into S-p*K*_a_ dataset.


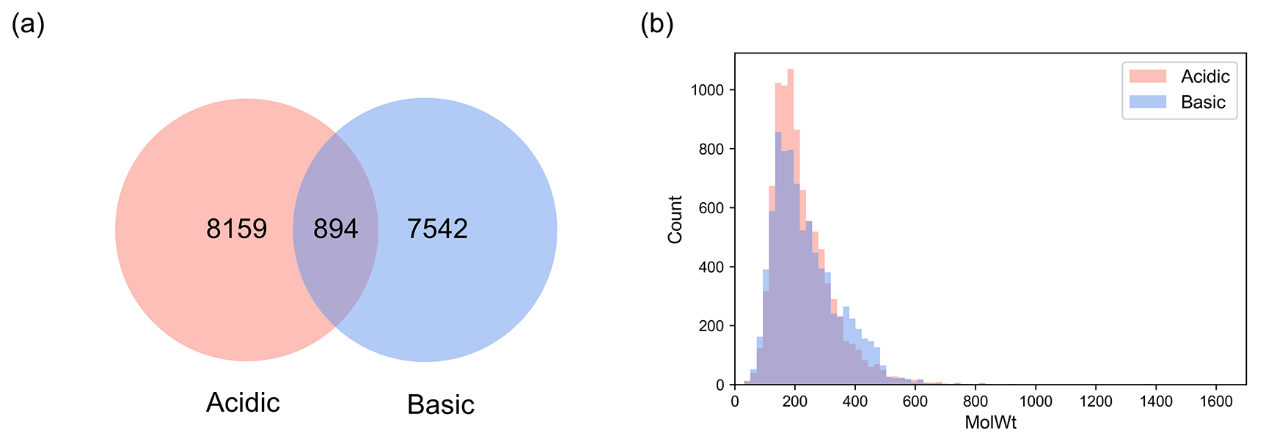
**Fig. S2.** (a) Venn diagram showing compounds with acidic or basic p*K*_a_ values. (d) The molecular weight distribution of compounds in the S-p*K*_a_ dataset.

**The derivation of** **formula 3 and 6**

**
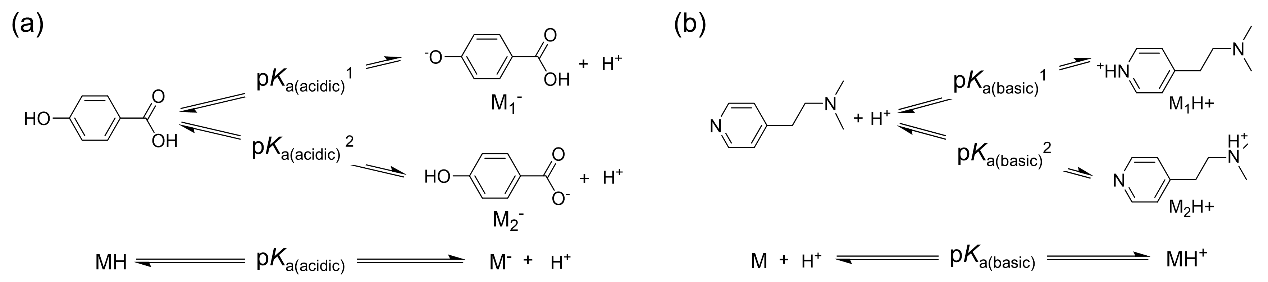
**

**Fig. S3.** Macro and micro ionization equilibrium equations of acidic (a) and basic (b) compounds.

For acidic compound in Fig. S3a, the equilibrium constant of micro ionization can be expressed as:

$$\begin{aligned} {K_{\left( \mathrm{acidic} \right)}}^{1}= \frac{\left[ \text{M}\text{1}\text{-} \right]*\left[ \text{H}\text{+} \right]}{\left[ \text{MH} \right]} \#\left( S1 \right) \end{aligned}$$

$$\begin{aligned} {K_{\left( \mathrm{acidic} \right)}}^{2}= \frac{\left[ \text{M}\text{2}\text{-} \right]*\left[ \text{H}\text{+} \right]}{\left[ \text{MH} \right]} \#\left( S2 \right) \end{aligned}$$

the equilibrium constant of macro ionization can be expressed as:

$$\begin{aligned} K_{\left( \mathrm{acidic} \right)}= \frac{\left[ \text{M}\text{-} \right]*\left[ \text{H}\text{+} \right]}{\left[ \text{MH} \right]} \#\left( S3 \right) \end{aligned}$$

$$\begin{aligned} K_{\left( \mathrm{acidic} \right)}= \frac{\left[ \text{M}\text{1}\text{-} \right]*\left[ \text{H}\text{+} \right]+\left[ \text{M}\text{2}\text{-} \right]*\left[ \text{H}\text{+} \right]}{\left[ \text{MH} \right]} \#\left( S4 \right) \end{aligned}$$

Substituting S1 and S2 in S4,

$$\begin{aligned} \text{K}\text{(acidic)}={K_{\left( \mathrm{acidic} \right)}}^{1}\text{+}{K_{\left( \mathrm{acidic} \right)}}^{2}\#\left( S5 \right) \end{aligned}$$

Taking negative logarithms in S5,

$$\begin{aligned} -\log\left( \text{K}\text{(acidic)} \right)=-log({K_{\left( \mathrm{acidic} \right)}}^{1}\text{+}{K_{\left( \mathrm{acidic} \right)}}^{2})\#\left( S6 \right) \end{aligned}$$

$$\begin{aligned} {pK}_{a\left( \mathrm{acidic} \right)}=-log({10}^{{{-pK}_{a\left( \mathrm{acidic} \right)}}^{1}}+{10}^{{{-pK}_{a\left( \mathrm{acidic} \right)}}^{2}})\#\left( S7 \right) \end{aligned}$$

For compound have *N* acidic ionizable sites,

$$\begin{aligned} {pK}_{a\left( \mathrm{acidic} \right)}=-\log\left( \sum_{i=1}^{N} {10}^{{{-pK}_{a\left( \mathrm{acidic} \right)}}^{i}} \right) \#\left( S8 \right) \end{aligned}$$

For basic compound in Fig. S3b, the equilibrium constant of micro ionization can be expressed as:

$$\begin{aligned} {K_{\left( \mathrm{basic} \right)}}^{1}= \frac{\left[ \text{M} \right]*\left[ \text{H}\text{+} \right]}{\left[ \text{M}\text{1}\text{H}\text{+} \right]} \#\left( S9 \right) \end{aligned}$$

$$\begin{aligned} {K_{\left( \mathrm{basic} \right)}}^{2}= \frac{\left[ \text{M} \right]*\left[ \text{H}\text{+} \right]}{\left[ \text{M}\text{2}\text{H}\text{+} \right]}\#\left( S10 \right) \end{aligned}$$

the equilibrium constant of macro ionization can be expressed as:

$$\begin{aligned} K_{\left( \mathrm{basic} \right)}= \frac{\left[ \text{M} \right]*\left[ \text{H}\text{+} \right]}{\left[ \text{MH}\text{+} \right]}\#\left( S11 \right) \end{aligned}$$

$$\begin{aligned} K_{\left( \mathrm{basic} \right)}= \frac{\left[ \text{M} \right]*\left[ \text{H}\text{+} \right]}{\left[ \text{M}\text{1}\text{H}\text{+} \right]+\left[ \text{M}\text{2}\text{H}\text{+} \right]} \#\left( S12 \right) \end{aligned}$$

Taking reciprocal in S12 as well as substituting S9 and S10 in S12,

$$\begin{aligned} \frac{1}{K_{\left( \mathrm{basic} \right)}}=\frac{1}{{K_{\left( \mathrm{basic} \right)}}^{1}} +\frac{1}{{K_{\left( \mathrm{basic} \right)}}^{2}}\#\left( S13 \right) \end{aligned}$$

Taking negative logarithms in S13,

$$\begin{aligned} -\log\left( \frac{1}{K_{\left( \mathrm{basic} \right)}} \right)=-\log(\frac{1}{{K_{\left( \mathrm{basic} \right)}}^{1}} +\frac{1}{{K_{\left( \mathrm{basic} \right)}}^{2}})\#\left( S14 \right) \end{aligned}$$

$$\begin{aligned} {-pK}_{a\left( \mathrm{basic} \right)}=-log({10}^{{{pK}_{a\left( \mathrm{basic} \right)}}^{1}}+{10}^{{{pK}_{a\left( \mathrm{basic} \right)}}^{2}})\#\left( S15 \right) \end{aligned}$$

For compound have *N* basic ionizable sites,

$$\begin{aligned} {pK}_{a\left( \mathrm{basic} \right)}=\log\left( \sum_{i=1}^{N} {10}^{{{pK}_{a\left( \mathrm{basic} \right)}}^{i}} \right)\#\left( S16 \right) \end{aligned}$$

As shown, for the compounds that only have acidic ionizable sites or basic ionizable sites, formulas S8 and S16 are strictly derived in describing the relationship between their most acidic/basic p*K*_a_ values and corresponding micro p*K*_a_ values. For the compounds that have both acidic ionizable sites and basic ionizable sites, formula S8 ignores the influence of basic ionizable sites in calculating the most acidic p*K*_a_, while formula S16 ignores the influence of acidic ionizable sites in calculating the most basic p*K*_a_. According to our tests, this simplification works fine for common amphoteric compounds such as amino acids and aminophenol.

**Table S1.** The initial atom and bond features

| atom feature | atom symbol | 43 | [C, N, O, S, F, Si, P, Cl, Br, Mg, Na, Ca, Fe, As, Al, I, B, V, K, Tl, Yb, Sb, Sn, Ag, Pd, Co, Se, Ti, Zn, H, Li, Ge, Cu, Au, Ni, Cd, In, Mn, Zr, Cr, Pt, Hg, Pb] (one-hot) |
| --- | --- | --- | --- |
|  | degree | 11 | number of covalent bonds (0-10, one-hot) |
|  | implicit hydrogens | 7 | number of implicit hydrogens (0-6 one-hot) |
|  | formal charge | 1 | electrical charge (integer) |
|  | radical electrons | 1 | number of radical electrons (integer) |
|  | hybridization | 5 | [sp, sp2, sp3, sp3d, sp3d2] (one-hot) |
|  | aromaticity | 1 | whether the atom is aromatic (one-hot) |
|  | hydrogens | 5 | number of connected hydrogens (0–4, one-hot) |
| bond feature | bond type | 4 | [single, double, triple, aromatic] (one-hot) |
|  | conjugation | 1 | whether the bond is conjugated |
|  | ring | 1 | whether the bond is in ring |
|  | stereo | 6 | [StereoNone, StereoAny, StereoZ, StereoE, StereoCIS, StereoANS] (one-hot) |

**Table S2.** The search ranges and optimal values of hyperparameters

| model | Hyperparameter | Possible values | optimal value |
| --- | --- | --- | --- |
| SVM | kernel | ‘linear’, ‘rdf’ | 'rdf' |
|  | gamma | 0.00001, 0.0001, 0.001, 0.01,0.1,1,10 | 0.001 |
|  | C | 0.1,1,10,100,1000,10000,100000 | 100 |
| RF | n_estimators | 30,50,70,100,150,300 | 100 |
|  | max_depth | 3,5,10,15,20,30, None | None |
|  | min_samples_split | 2,3,4,5 | 2 |
| ANN | Layer_size | 50,100,200,400,800 | 200(acid)  800(base) |
|  | Layer_num | 2,3,4,5 | 4(acid)  3(base) |
|  | alpha | 0.0001,0.001 | 0.0001 |
|  | learning_rate_init | 0.0001, 0.001, 0.01 | 0.001 |
| XGBoost | n_estimators | 50,100,300,500,1000,2000,3000 | 2000 |
|  | learning_rate | 0.1,0.2,0.5 | 0.1 |
|  | max_depth | 3,4,6,9 | 6 |
|  | subsample | 1.0,0.9,0.8,0.5 | 0.8 |
| Attentive FP | Layer_num | 3,4,5,6,7 | 6 |
|  | num_timesteps | 1,2,3 | 1 |
|  | Layer_size | 100,200,400 | 200 |
|  | weight_decay | 0.0003,0.003 | 0.0003 |
|  | learning_rate | 0.0003, 0.00003 | 0.0003 |
| Graph-p*K*_a_ | Layer_num | 3,4,5,6,7 | 6 |
|  | Layer_size | 100,200,400 | 200 |
|  | weight_decay | 0.0003,0.003 | 0.0003 |
|  | learning_rate | 0.0003, 0.00003 | 0.0003 |

**Table S3.** The number of molecules on various similarity subsets

| Test set | maximum similarity to training set molecules | number of molecules |
| --- | --- | --- |
| Acidic | 0.8-1 | 258 |
|  | 0.7-0.8 | 203 |
|  | 0.6-0.7 | 328 |
|  | 0.5-0.6 | 312 |
|  | 0-0.5 | 257 |
| Basic | 0.8-1 | 304 |
|  | 0.7-0.8 | 175 |
|  | 0.6-0.7 | 264 |
|  | 0.5-0.6 | 284 |
|  | 0-0.5 | 239 |

**Table S4.** The predicted results of Graph-p*K*_a_ and other models on the SAMPL6 and SAMPL7 external test sets

| SMILES | Molecule ID | p*K*_a_ ID | Source | p*K*_a_ (exp) | p*K*_a_ class | Graph-p*K*_a_  1 | Graph-p*K*_a_  2 | Graph-p*K*_a_  3 | Epik Scan | Epik Microscopic | ACD/ pKa classic | MoKa | ChemAxon | Hunt’s model | Yang’s XGBoost | Yang’s  Neural Network | OPERA |
| --- | --- | --- | --- | --- | --- | --- | --- | --- | --- | --- | --- | --- | --- | --- | --- | --- | --- |
| O=C1NCCCc2c1oc1ccc(O)cc21 | SM01 | SM01_1 | SAMPL6 | 9.53 | most acidic | 8.670529 | 8.631477 | 8.379063 | 9.12 | 9.12 | 9 | 10.72 | 9.44 | 10.28 | 9.41 | 8.58 | 6.97 |
| FC(F)(F)c1cccc(Nc2ncnc3ccccc23)c1 | SM02 | SM02_1 | SAMPL6 | 5.03 | most basic | 3.834098 | 4.233867 | 4.25936 | 4.05 | 4.16 | 4.52 | 4.89 | 4.01 | 5.349 | 3.95 | 3.68 | 6.16 |
| O=C(Nc1nnc(Cc2ccccc2)s1)c1cccs1 | SM03 | SM03_1 | SAMPL6 | 7.02 | most acidic | 7.825701 | 7.473364 | 7.537795 | 7.12 | 7.12 | 8.17 | 6.08 | 7.68 | 4.933 | 9.02 | 8.35 | 6.89 |
| Clc1ccc(CNc2ncnc3ccccc23)cc1 | SM04 | SM04_1 | SAMPL6 | 6.02 | most basic | 4.930722 | 5.548564 | 5.221679 | 5.6 | 5.61 | 5.13 | 5.11 | 4.83 | 4.879 | 5.38 | 4.73 | 6.94 |
| O=C(Nc1ccccc1N1CCCCC1)c1ccc(Cl)o1 | SM05 | SM05_1 | SAMPL6 | 4.59 | most basic | 4.591392 | 4.558191 | 4.679179 | 5.35 | 5.35 | 5.77 | 3.51 | 4.19 | 6.375 | 6.58 | 4.22 | 2.8 |
| O=C(Nc1cccc2cccnc12)c1cncc(Br)c1 | SM06 | SM06_1 | SAMPL6 | 3.03 | most basic | 3.862032 | 3.100895 | 3.761634 | 2.18 | 2.17 | 1.97 | 4.08 | 3.42 | 2.224 | 3.85 | 3.63 | 3.82 |
| O=C(Nc1cccc2cccnc12)c1cncc(Br)c1 | SM06 | SM06_2 | SAMPL6 | 11.74 | most acidic | 11.97671 | 11.26938 | 11.74034 | 10.33 | 10.33 | 10.84 | 10.57 | 13.85 | 11.5 | 10.78 | 10.19 | NaN |
| c1ccc(CNc2ncnc3ccccc23)cc1 | SM07 | SM07_1 | SAMPL6 | 6.08 | most basic | 4.93092 | 5.558676 | 5.221679 | 5.6 | 5.61 | 5.51 | 5.11 | 4.83 | 4.981 | 5.32 | 5.33 | 6.73 |
| Cc1ccc2[nH]c(=O)c(CC(=O)O)c(-c3ccccc3)c2c1 | SM08 | SM08_1 | SAMPL6 | 4.22 | most acidic | 3.805479 | 4.013884 | 4.198157 | 4.11 | 4.11 | 3.93 | 2.75 | 4.2 | 4.459 | 4.76 | 4.87 | 3.17 |
| COc1cccc(Nc2ncnc3ccccc23)c1 | SM09 | SM09_1 | SAMPL6 | 5.37 | most basic | 3.974639 | 4.429934 | 4.260782 | 4.05 | 4.16 | 4.78 | 4.89 | 4 | 5.023 | 5.17 | 5.36 | 6.08 |
| O=C(CNC(=O)c1ccccc1)Nc1nc2ccccc2s1 | SM10 | SM10_1 | SAMPL6 | 9.02 | most acidic | 9.641075 | 10.41825 | 9.795728 | 8.67 | 8.67 | 9.16 | 11.16 | 7.77 | 10.11 | 9.5 | 8.79 | 9.34 |
| Nc1ncnc2c1cnn2-c1ccccc1 | SM11 | SM11_1 | SAMPL6 | 3.89 | most basic | 3.830474 | 3.878787 | 3.628914 | 3.87 | 3.87 | 3.93 | 4.23 | 3.42 | 3.761 | 3.91 | 4 | 3.79 |
| Clc1cccc(Nc2ncnc3ccccc23)c1 | SM12 | SM12_1 | SAMPL6 | 5.28 | most basic | 3.879416 | 4.275062 | 4.256599 | 4.05 | 4.16 | 4.44 | 4.89 | 4.01 | 4.585 | 4.06 | 4.08 | 5.07 |
| COc1cc2ncnc(Nc3cccc(C)c3)c2cc1OC | SM13 | SM13_1 | SAMPL6 | 5.77 | most basic | 5.066304 | 4.779203 | 5.032618 | 4.27 | 4.44 | 5.01 | 5.64 | 4.68 | 5.393 | 5.69 | 5.89 | 3.57 |
| Nc1ccc2c(c1)ncn2-c1ccccc1 | SM14 | SM14_2 | SAMPL6 | 5.3 | most basic | 5.347182 | 4.816226 | 5.603992 | 6.35 | 6.35 | 5.16 | 6.09 | 6.4 | 4.383 | 5.55 | 5.02 | 3.92 |
| Oc1ccc(-n2cnc3ccccc32)cc1 | SM15 | SM15_1 | SAMPL6 | 4.7 | most basic | 4.897919 | 4.172761 | 4.757471 | 5.82 | 5.82 | 4.78 | 4.95 | 4.91 | 5.368 | 4.9 | 4.9 | NaN |
| Oc1ccc(-n2cnc3ccccc32)cc1 | SM15 | SM15_2 | SAMPL6 | 8.94 | most acidic | 9.602269 | 8.812258 | 9.419638 | 8.71 | 8.71 | 9.07 | 9.39 | 10.64 | 8.957 | 8.58 | 8.92 | 9.95 |
| O=C(Nc1ccncc1)c1c(Cl)cccc1Cl | SM16 | SM16_1 | SAMPL6 | 5.37 | most basic | 5.256861 | 5.350735 | 5.471334 | 4.71 | 4.71 | 5.19 | 5.61 | 5.61 | 5.779 | 3.91 | 3.35 | 4.25 |
| O=C(Nc1ccncc1)c1c(Cl)cccc1Cl | SM16 | SM16_2 | SAMPL6 | 10.65 | most acidic | 12.04911 | 11.94477 | 12.45475 | 9.85 | 9.85 | 10.85 | 10.81 | 13.98 | 11.37 | 11.21 | 10.42 | NaN |
| c1ccc(CSc2nnc(-c3ccncc3)o2)cc1 | SM17 | SM17_1 | SAMPL6 | 3.16 | most basic | 3.463161 | 3.291993 | 3.57061 | 4.9 | 4.9 | 0.99 | 5.27 | 2.48 | 3.325 | 3.26 | 3.68 | NaN |
| O=C(CCc1nc2ccccc2c(=O)[nH]1)Nc1ncc(Cc2ccc(F)c(F)c2)s1 | SM18 | SM18_1 | SAMPL6 | 2.15 | most basic | 2.771059 | 2.835245 | 3.116422 | 2.48 | 2.23 | 2.34 | 2.42 | 1.55 | 3.636 | 3.28 | 3.7 | NaN |
| O=C(CCc1nc2ccccc2c(=O)[nH]1)Nc1ncc(Cc2ccc(F)c(F)c2)s1 | SM18 | SM18_2 | SAMPL6 | 9.58 | most acidic | 9.807236 | 9.283881 | 9.472614 | 9.38 | 9.16 | 9.39 | 8.6 | 8.04 | 9.064 | 6.96 | 5.9 | 6.18 |
| CCOc1ccc2nc(NC(=O)Cc3ccc(Cl)c(Cl)c3)sc2c1 | SM19 | SM19_1 | SAMPL6 | 9.56 | most acidic | 9.935365 | 10.13415 | 9.965655 | 9.17 | 9.17 | 9.91 | 9.97 | 7.89 | 9.969 | 9.61 | 8.77 | 9.85 |
| O=C1NC(=O)/C(=C\c2cccc(OCc3ccc(Cl)cc3Cl)c2)S1 | SM20 | SM20_1 | SAMPL6 | 5.7 | most acidic | 5.174829 | 4.618419 | 6.481783 | 8.05 | 8.05 | 6.01 | 6.76 | 7.2 | 7.228 | 6.98 | 6.23 | 5.29 |
| Fc1cnc(Nc2cccc(Br)c2)nc1Nc1cccc(Br)c1 | SM21 | SM21_1 | SAMPL6 | 4.1 | most basic | 3.598081 | 3.245317 | 3.323534 | 3.89 | 3.9 | 4.24 | 5.41 | 1.95 | 3.985 | 4.36 | 2.36 | 4.15 |
| Oc1c(I)cc(I)c2cccnc12 | SM22 | SM22_1 | SAMPL6 | 2.4 | most basic | 3.598081 | 2.64654 | 2.64791 | 3.56 | 3.5 | 2.43 | 2 | 3.34 | 1.92 | 2.94 | 2.3 | NaN |
| Oc1c(I)cc(I)c2cccnc12 | SM22 | SM22_2 | SAMPL6 | 7.43 | most acidic | 7.877353 | 8.090374 | 8.317771 | 6.79 | 6.79 | 7.59 | 7.66 | 7.39 | 8.316 | 7.71 | 6.23 | 7.9 |
| CCOC(=O)c1ccc(Nc2cc(C)nc(Nc3ccc(C(=O)OCC)cc3)n2)cc1 | SM23 | SM23_1 | SAMPL6 | 5.45 | most basic | 5.473255 | 5.10014 | 5.91773 | 5.99 | 6 | 5.36 | 6.06 | 5.02 | 5.889 | 4.78 | 4.94 | 4.79 |
| COc1ccc(-c2oc3ncnc(NCCO)c3c2-c2ccc(OC)cc2)cc1 | SM24 | SM24_1 | SAMPL6 | 2.6 | most basic | 3.509475 | 3.339419 | 4.033972 | 3.99 | 0.64 | 4.74 | 4.11 | 3.09 | 2.54 | 4.18 | 2.86 | NaN |
| O=C(CCc1ccccc1)NS(=O)(=O)c1ccccc1 | SM25 | SM25_1 | SAMPL7 | 4.49 | most acidic | 4.693657 | 4.431005 | 4.755572 | 5.49 |  |  |  | 4.24 |  | 6.32 | 6.1 | 7.05 |
| CC(=O)NS(=O)(=O)CCc1ccccc1 | SM26 | SM26_1 | SAMPL7 | 4.91 | most acidic | 4.951441 | 4.878375 | 5.081221 | 5.32 |  |  |  | 4.09 |  | 6.64 | 7.08 | 7.51 |
| CC1(NS(=O)(=O)CCc2ccccc2)COC1 | SM27 | SM27_1 | SAMPL7 | 10.45 | most acidic | 12.19729 | 10.46268 | 10.79291 | 11.22 |  |  |  | 9.59 |  | 9.23 | 11.03 | 10.06 |
| CS(=O)(=O)NC1(CCc2ccccc2)COC1 | SM29 | SM29_1 | SAMPL7 | 10.05 | most acidic | 12.22824 | 10.51957 | 11.20606 | 11.12 |  |  |  | 9.58 |  | 9.27 | 10.58 | 8.39 |
| O=S(=O)(NC1(CCc2ccccc2)COC1)c1ccccc1 | SM30 | SM30_1 | SAMPL7 | 10.29 | most acidic | 11.54214 | 9.740994 | 10.08566 | 10.56 |  |  |  | 10.15 |  | 9.44 | 11.15 | 9.31 |
| CN(C)S(=O)(=O)NC1(CCc2ccccc2)COC1 | SM31 | SM31_1 | SAMPL7 | 11.02 | most acidic | 12.50579 | 11.18729 | 11.86512 | 7.72 |  |  |  | 9.78 |  | 8.81 | 13.15 | 8.78 |
| CS(=O)(=O)NC1(CCc2ccccc2)CSC1 | SM32 | SM32_1 | SAMPL7 | 10.45 | most acidic | 12.00724 | 10.36085 | 11.23481 | 11.17 |  |  |  | 10.7 |  | 9.07 | 10.34 | 7.31 |
| CN(C)S(=O)(=O)NC1(CCc2ccccc2)CSC1 | SM34 | SM34_1 | SAMPL7 | 11.93 | most acidic | 12.33558 | 11.04397 | 11.86678 | 7.77 |  |  |  | 10.89 |  | 8.84 | 11.15 | 6.77 |
| CS(=O)(=O)NC1(CCc2ccccc2)C[S+]([O-])C1 | SM35 | SM35_1 | SAMPL7 | 9.87 | most acidic | 11.11037 | 10.60809 | 8.288017 | 10.44 |  |  |  | 10.16 |  | 8.2 | 9.67 | 6.91 |
| O=S(=O)(NC1(CCc2ccccc2)C[S+]([O-])C1)c1ccccc1 | SM36 | SM36_1 | SAMPL7 | 9.8 | most acidic | 10.30419 | 10.1775 | 7.799403 | 9.87 |  |  |  | 10.16 |  | 8.61 | 10.14 | 8.02 |
| CN(C)S(=O)(=O)NC1(CCc2ccccc2)C[S+]([O-])C1 | SM37 | SM37_1 | SAMPL7 | 10.33 | most acidic | 11.22263 | 11.02243 | 8.490375 | 7.04 |  |  |  | 10.42 |  | 8.11 | 10.41 | 6.23 |
| CS(=O)(=O)NC1(CCc2ccccc2)CS(=O)(=O)C1 | SM38 | SM38_1 | SAMPL7 | 9.44 | most acidic | 10.15964 | 10.53941 | 9.392019 | 10.25 |  |  |  | 9.07 |  | 9 | 8.66 | 7 |
| O=S1(=O)CC(CCc2ccccc2)(NS(=O)(=O)c2ccccc2)C1 | SM39 | SM39_1 | SAMPL7 | 10.22 | most acidic | 9.264742 | 9.694185 | 8.794205 | 9.69 |  |  |  | 10.16 |  | 8.5 | 8.95 | 8.01 |
| CN(C)S(=O)(=O)NC1(CCc2ccccc2)CS(=O)(=O)C1 | SM40 | SM40_1 | SAMPL7 | 9.58 | most acidic | 9.990362 | 11.13107 | 9.923714 | 6.85 |  |  |  | 9.35 |  | 8.3 | 9.26 | 5.92 |
| CS(=O)(=O)Nc1cc(-c2ccccc2)on1 | SM41 | SM41_1 | SAMPL7 | 5.22 | most acidic | 5.937761 | 6.06148 | 5.869885 | 5.22 |  |  |  | 6.44 |  | 7.94 | 5.88 | 6.38 |
| O=S(=O)(Nc1cc(-c2ccccc2)on1)c1ccccc1 | SM42 | SM42_1 | SAMPL7 | 6.62 | most acidic | 5.127365 | 5.377483 | 5.435533 | 5 |  |  |  | 5.82 |  | 7.97 | 6.06 | 7.04 |
| CN(C)S(=O)(=O)Nc1cc(-c2ccccc2)on1 | SM43 | SM43_1 | SAMPL7 | 5.62 | most acidic | 6.235569 | 6.810569 | 6.609465 | 5.04 |  |  |  | 7.17 |  | 6.9 | 5.93 | 5.43 |
| CS(=O)(=O)Nc1cn(-c2ccccc2)nn1 | SM44 | SM44_1 | SAMPL7 | 6.34 | most acidic | 5.721827 | 5.648724 | 6.356681 | 6.27 |  |  |  | 6.48 |  | 6.95 | 4.37 | NaN |
| O=S(=O)(Nc1cn(-c2ccccc2)nn1)c1ccccc1 | SM45 | SM45_1 | SAMPL7 | 5.93 | most acidic | 4.819913 | 4.916687 | 5.914379 | 6.05 |  |  |  | 5.72 |  | 7.03 | 4.36 | NaN |
| CN(C)S(=O)(=O)Nc1cn(-c2ccccc2)nn1 | SM46 | SM46_1 | SAMPL7 | 6.42 | most acidic | 6.274833 | 6.523676 | 7.340707 | 6.09 |  |  |  | 7.2 |  | 5.56 | 4.62 | 7.2 |

**
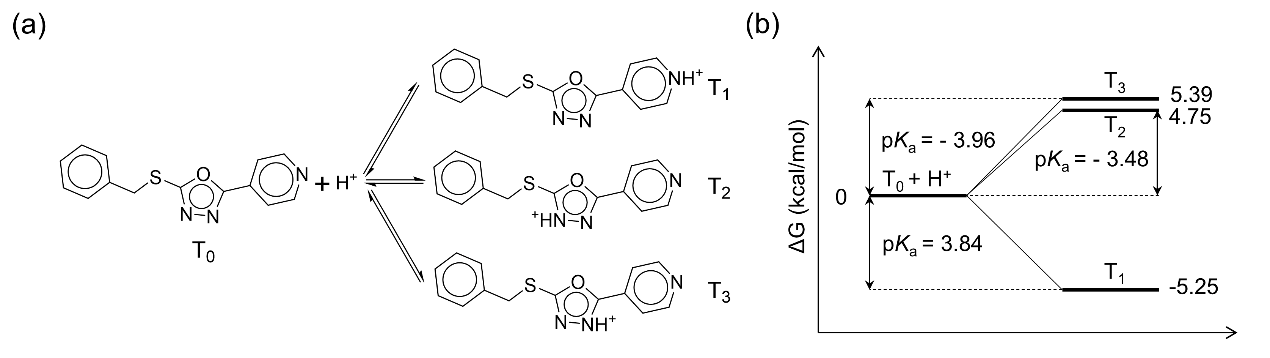
Fig. S4.** (a) The dissociation scheme of molecule B2. (b) The relative energy between various protonation states of molecule B2 calculated by quantum chemical calculation.

**The method of quantum chemistry calculation**

The method of quantum chemistry calculation was referred to a previous study [25]. All the DFT calculations were carried out with the Gaussian 16 package. Geometry optimizations were performed with B3LYP [26-28] functional with the 6-311G(d,p) [29] basis set. Frequency analysis was conducted at the same level of theory to verify the stationary points to be minimum or saddle points. The single-point energies were calculated using B3LYP functional with the def2-TZVP basis set [30]. The solvent effects were computed by using the SMD solvation model (solvent=water) in all DFT calculations.


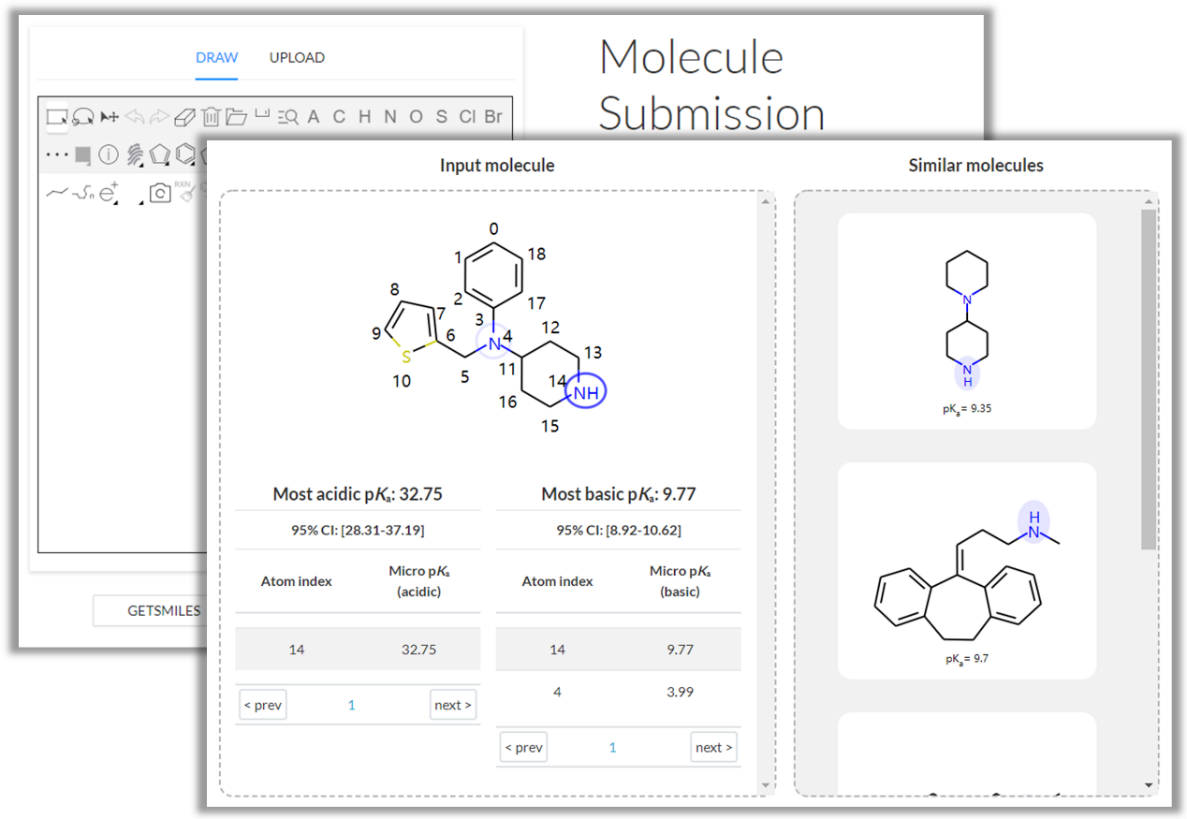


**Fig. S5.** Snapshot of the web-server input and output interface of Graph-p*K*_a_, which contains the illustration of molecule submission, p*K*_a_ prediction, and similarity search results.

**Reference**

[1] Hunt PA, Hosseini-Gerami L, Chrien T, et al. Predicting pka using a combination of semi-empirical quantum mechanics and radial basis function methods. J Chem Inf Model 2020; 60: 2989–2997.

[2] Manallack DT. The pka distribution of drugs: Application to drug discovery. Perspect Med Chem 2007; 1: 25-28.

[3] Milletti F, Storchi L, Sforna G, et al. New and original pka prediction method using grid molecular interaction fields. J Chem Inf Model 2007; 47: 2172-2181.

[4] Bagno A, Lovato G, Scorrano G. Thermodynamics of protonation and hydration of aliphatic amides. J Chem Soc, Perkin Trans 2 1993; 1091-1098.

[5] Souza BS, Mora JR, Wanderlind EH, et al. Transforming a stable amide into a highly reactive one: Capturing the essence of enzymatic catalysis. Angew Chem, Int Ed 2017; 129: 5429-5432.

[6] Cox RA. Lactams in sulfuric acid. The mechanism of amide hydrolysis in weak to moderately strong aqueous mineral acid media. Can J Chem 1998; 76: 649-656.

[7] Katritzky A, Waring A, Yates K. The hammett acidity function and its non-applicability to amides. Tetrahedron 1963; 19: 465-469.

[8] Joule JA, Mills K. Heterocyclic chemistry. John Wiley & Sons, 2008

[9] Stewart R, Dolman D. A comparison of the acidity and basicity of aromatic amines. Can J Chem 1967; 45: 925-928.

[10] Milletti F, Storchi L, Goracci L, et al. Extending pka prediction accuracy: High-throughput pka measurements to understand pka modulation of new chemical series. Eur J Med Chem 2010; 45: 4270-4279.

[11] Dardonville C, Caine BA, de la Fuente MN, et al. Substituent effects on the basicity (pka) of aryl guanidines and 2-(arylimino) imidazolidines: Correlations of ph-metric and uv-metric values with predictions from gas-phase ab initio bond lengths. New J Chem 2017; 41: 11016-11028.

[12] Lõkov M, Tshepelevitsh S, Heering A, et al. On the basicity of conjugated nitrogen heterocycles in different media. 2017;

[13] Yu H, Wondrousch D, Yuan Q, et al. Modeling and predicting pka values of mono-hydroxylated polychlorinated biphenyls (ho-pcbs) and polybrominated diphenyl ethers (ho-pbdes) by local molecular descriptors. Chemosphere 2015; 138: 829-836.

[14] Thapa B, Schlegel HB. Improved pka prediction of substituted alcohols, phenols, and hydroperoxides in aqueous medium using density functional theory and a cluster-continuum solvation model. J Phys Chem A 2017; 121: 4698-4706.

[15] Wuitschik G, Rogers‐Evans M, Müller K, et al. Oxetanes as promising modules in drug discovery. Angew Chem, Int Ed 2006; 45: 7736-7739.

[16] Method for producing carbamate compound. 2016, CN106008270A.

[17] Marín-Luna M, Alkorta I, Elguero J. A theoretical study of the gas phase (proton affinity) and aqueous (pka) basicity of a series of 150 pyrazoles. New J Chem 2015; 39: 2861-2871.

[18] Mertsalov S, Egorova L, Postovskii IY. Benzodiazines. Chem Heterocycl Compds 1970; 6: 636-639.

[19] Caine BA, Bronzato M, Popelier PL. Experiment stands corrected: Accurate prediction of the aqueous pka values of sulfonamide drugs using equilibrium bond lengths. Chem Sci 2019; 10: 6368-6381.

[20] Qiang Z, Adams C. Potentiometric determination of acid dissociation constants (pka) for human and veterinary antibiotics. Water Res 2004; 38: 2874-2890.

[21] Zheng Y, Zheng W, Zhu D, et al. Theoretical modeling of p k a's of thiol compounds in aqueous solution. New J Chem 2019; 43: 5239-5254.

[22] Settimo L, Bellman K, Knegtel RM. Comparison of the accuracy of experimental and predicted pka values of basic and acidic compounds. Pharm Res 2014; 31: 1082-1095.

[23] Baltruschat M, Czodrowski P. Machine learning meets pka. F1000Research 2020; 9:

[24] Mansouri K, Cariello NF, Korotcov A, et al. Open-source qsar models for pka prediction using multiple machine learning approaches. J Cheminf 2019; 11: 60.

[25] Bochevarov AD, Watson MA, Greenwood JR, et al. Multiconformation, density functional theory-based pka prediction in application to large, flexible organic molecules with diverse functional groups. J Chem Theory Comput 2016; 12: 6001-6019.

[26] Lee C, Yang W, Parr RG. Development of the colle-salvetti correlation-energy into a function of the electron density. Phys Rev B 1988; 37: 785-789.

[27] Becke AD. A new mixing of hartree–fock and local density‐functional theories. J Chem Phys 1993; 98: 1372-1377.

[28] Stephens PJ, Devlin FJ, Chabalowski CF, et al. Ab initio calculation of vibrational absorption and circular dichroism spectra using density functional force fields. J Chem Phys 1994; 98: 11623-11627.

[29] Hehre WJ, Ditchfield R, Pople JA. Self—consistent molecular orbital methods. Xii. Further extensions of gaussian—type basis sets for use in molecular orbital studies of organic molecules. J Chem Phys 1972; 56: 2257-2261.

[30] Weigend F, Ahlrichs R. Balanced basis sets of split valence, triple zeta valence and quadruple zeta valence quality for h to rn: Design and assessment of accuracy. Phys Chem Chem Phys 2005; 7: 3297-3305.
